# Phantom Limb Sensation (PLS) and Phantom Limb Pain (PLP) among Young Landmine Amputees

**Published:** 2016

**Authors:** Mahtab POOR ZAMANY NEJATKERMANY, Ehsan MODIRIAN, Mohammadreza SOROUSH, Mehdi MASOUMI, Maryam HOSSEINI

**Affiliations:** 1 Anesthesiology and Critical Care Deprtment, Labbafinejad Hospital, Medical University of ShahidBeheshti, Tehran, Iran; 2 Emergency Medicine Department, Boali Hospital, Qazvin University of Medical Sciences, Qazvin, Iran; 3 Janbazan Medical and Engineering Research Center (JMERC), Tehran, Iran

**Keywords:** Amputation, Phantom pain, Phantom sensation, War

## Abstract

**Objective:**

To determine the frequency of phantom limb sensation (PLS) and phantom limb pain (PLP) in children and young adults suffering landmine-related amputation.

**Materials & Methods:**

All youths with amputation due to landmine explosions participated in this study. The proportions of patients with phantom limb sensation/pain, intensity and frequency of pain were reported. Chi square test was used to examine the relationship between variables. Comparison of PLP and PLS between upper and lower amputation was done by unpaired t-test.

**Results:**

There were 38 male and 3 female with the mean age of 15.8±2.4yr. The mean interval between injury and follow-up was 90.7±39.6 months. Twelve (44.4%) upper limb amputees and 11 (26.8%) lower limb amputees had PLS. Nine (33.3%) upper limb amputees and 7 (17.1%) lower limb amputees experienced PLP. Of 27 upper limb amputees, 6 (14.6%) and among 15 lower limb amputees, 6 (14.6%) had both PLS and PLP. One case suffered amputation of upper and lower limbs and was experiencing PLS and PLP in both parts. PLS had a significant difference between the upper and lower amputated groups. Significant relationship was observed between age of casualty and duration of injury with PLP.

**Conclusion:**

Phantom limb sensation and pain in young survivors of landmine explosions appear to be common, even years after amputation.

## Introduction

Following amputation, individuals usually perceive pain in their missing limb. Phantom limb sensation (PLS) is feeling of a lost body part after traumatic injuries (1). It is often painful (PLP) and a common experience among amputees (2). In case of etiology and mechanism of phantom limb pain, different theories have been proposed over the years. At first, it was thought to be a psychiatric illness but in the base of the different theories, pain was severed nerve ending and formation of neuroma. In recent years, phantom pain is considered to be changes in the peripheral and central nervous system. However, none of these theories canexplain PLP clearly and a combination of mechanisms is responsible (1). Presumably, the sensations are due to the brain’s tries to rearrange sensory information of lost limb. It is vivid feelings of posture and movement particularly in first days after surgery. In majority of cases, PLP occurs during the first years after amputation and disappears over time (3-4). PLS belonging amputations above the knee is stronger than below the knee (1). The majority of PLS do not require treatment and will recover within 2 to 3 yr but if it remain, phantom pain will appear (5). Compared to lower limb amputees, PLP is more prevalent in upper limb amputees (6-7). In lower limb amputees, the frequency and duration of the phantom pain will decrease remarkably but the intensity of pain will be constant (8). Conflicting reports have been published on the prevalence of phantom pain in children. The reported incidences of PLP vary from 47%-79% (9-11). A retrospective study reported PLP in child and adolescent amputees near to 40% (12), while, its incidence in same age group with amputation was reported as 3.7% (13). Besides, in amputees who underwent amputation before the age of 4 yr, PLP and PLS are rare. The incidence of phantoms increases follow amputation at or above 8yr (14). PLP is very common among amputee war veterans and mostly remain for years after amputation (15). One fifth of Iranian bilateral upper limb amputee war veterans with mean of 17 yr after trauma experienced PLS, and more than half suffered from PLP (16). One of the most frequent reasons for amputation in healthy people after end of wars is landmine explosion. The number of child casualties of landmines and unexploded ordnance (UXO) in 2010 was estimated about 1200 and children accounted for more than half of all civilian deaths (17). Generally, children are more interested to handle explosive devices and the blasts will cause injuries that usually result in amputations. There have been only a few studies on the subject of phantom pain and sensation in children and adolescents. Our aim was to investigate the existing of PLS and PLP in youth amputees due to postwar landmines explosions in Iran.

## Materials & Methods

Observations were made on whole of 41 survivors less than 18 yr old who had suffered amputation caused by landmine explosion in Iran. The study conducted by Janbazan Medical and Engineering Research Center (JMERC) of Iran, evaluated the prevalence of PLP among adolescents and children with amputations. All have been affected by landmines and UXO after armistice of Iran-Iraq war in 1988. The study group reported the intensity of their pain or pain level at presentation, using the verbal descriptor scale (VDS) included “least possible pain” and “worst possible pain”. Follow-up were assessed at least 36 and maximum 180 months after amputation. Moreover, demographic information obtained from the direct interview included age, sex, age of amputation, duration of injury, living area and type of activity at explosion time. Pain specialist directly examined all subjects. Cases were included if they had any type of amputations in upper and lower limbs or both. If pain was present, the location, severity and persistency would be asked. Statistical analyses were performed using SPSS 22.0 (Chicago, IL, USA). Data are expressed as means (SD) or numbers and percentage. The proportion of patients with PLS and PLP was reported. Relationship between variables was evaluated using chi-square test. Unpaired t-test (two tailed) was employed for comparison of PLP and PLS between upper and lower amputated subjects. A P value < 0.05 considered statistically significant. 

## Results

By the time of study, 41 amputees less than 18 yr old, consisted of 38 males (92.7%) and 3 females (7.3%), were identified. Twenty six (63.4%) had only upper limb amputation, 14 (34.2%) had only lower limb amputation and one (2.4%) had both. Three cases had bilateral upper limb amputation and one had bilateral lower limb amputation. Thus, there were 30 upper limb amputations and 16 lower limb amputations (27 subjects had upper limb amputations and 15 had lower limb amputations). Finger level (31.7%) and below the knee (17.1%) were the most common upper and lower limb amputations, respectively. Amputated limbs are listed in Table 1. Subjects’ age at the time of amputation ranged from 2 to 15 yr (mean, 8.2±3.3 yr). The mean age at the timeof follow-up was 15.8±2.4, ranged 9-18 yr and the mean number of months between casualty and followup were 90.7±39.6 months. Survivors had come from the five provinces of the western border of Iran as follows: 13 (31.7%) from Kurdistan and 13 (31.7%) from Kermanshah, 7 (17.1%) from West Azerbaijan, 6 (14.6%) from Ilam and 2 (4.9%) from Khuzestan. Besides, 23 (56.1%) of them lived in villages and 18 (43.9%) lived in cities. At the time of the casualties they were involved in such activities; 17 (41.5%) playing, 17 (41.5%) grazing livestock, 3 (7.3%) crossing place, 2 (4.9%) collecting food, 1 (2.4%) manipulation and 1 (2.4%) farming. Of cases with upper limb amputated, 6 (22.2%) experienced PLS, 3 (11.1%) reported PLP and 6 (22.2%) had both PLP and PLS. Among the subjects with lower limb amputated, PLS was reported by 5 (33.3%), PLP experienced by 1 (6.7%) and 6 (40.0%) had both PLP and PLS. Of those with PLP, 4 (9.8%) experienced constant or daily pain, 7 (17.1%) reported having pain almost always and 4 (9.8%) had pain occasionally. The only patient suffered upper and lower limb amputation had almost always PLS and distressing PLP. Moreover, phantom phenomena were reported for only one (2.44%) who lost upper extremity before the age of 5 yr with severity of distressing. The intensity of pain in their residual limbs is presented in Table 2. Comparison analysis showed PLS had a significant difference between the upper and lower amputated group (P=0.01). Anyway, there was no significant difference between them in PLP prevalence. On the other, there were direct correlations between age of casualty (P=0.04) and also duration of injury (P=0.03) with PLP. As well, no significant difference was observed in the severity and persisting of pain between upper and lower limb amputees.

## Discussion

Considering our purpose to determine the incidence of phantom limb phenomena among youth amputees who had undergone amputations due to landmine explosions, all of Iranian survivors less than eighteen years included in this study. Similar to many affected countries that more than half of the civilian victims and more than four fifth of child casualties of mine explosions areboys, almost all of participants were male (17). About two third of cases lost upper extremities raised out of activities like playing with or handling landmines assuming they are toys. Despite the double frequency of upper limb amputation, PLS and PLP were more common in lower limb amputees similar to former Iranian study (18). However, in comparison with lower limb amputations, phantom pain is more prevalent in upper limb amputations (6-7, 19). Assessing phantom limb pain in young cancer-related lower limb amputees, although 76% of patients had experienced PLP at first year after surgery, after the lapse of one year, only one in ten reported phantom pain (20). Besides, about two third of patients who incurred lower limb amputation had PLS, a quarter suffered PLP and one tenth experienced both after almost 17 months (21). Nevertheless, in this study we concluded that despite passing a long time since the casualties, more than half experienced PLS and PLP. Our finding are similar to that of a study reported the prevalence of one fifth of phantoms in the congenital limb deficient group and two fifth for young amputees who underwent amputation before the age of 6 yr (22). Our results are in consistent with a study which assessed prevalence of phantom sensations and pain in child and adolescent amputees aged 8 to 18 yr whom their amputations had been caused by congenital limb deficiency and surgery or trauma (13). However, in almost all studies of amputated children, diseases are the main cause of amputations while, our cases have experienced landmine explosions leading to amputations. Our findings are similar to some Iranian studies of adult amputee war veterans in which about half of the cases reported PLP and PLS more prevalent than PLP (16, 23-24). In investigation of the psychological effects of deficiencies on adolescent patients with limb amputation because of cancer, better adjustment to amputation was observed in amputees due to malignancy than traumatic ones (25). In a case study, a 15-yr-old girl with leg amputation was not experienced PLS consistently, but as discreet episodes. Moreover, decrease in duration and frequency of episodes and intensity of PLP was reported over one month (26). In the present study, about one-third of cases with PLP and two-third of upper limb amputees with PLP, were suffering distressing phantom pain. 

**Table1:** List of Amputated limbs in landmine explosion survivors under 18 years old

Amputation level	Right n (%)	Left n(%)	Both n(%)
Upper (n=27)			
** Fingers** ** or partial hand (Transcarpal)**	5 (18.5)	6 (22.2)	2 (7.4)
**At the wrist (Wrist disarticulation)**	4 (14.8)	2 (7.4)	0
**Below the elbow (Trans radial)**	0	0	0
**At the elbow (Elbow disarticulation)**	5 (18.5)	2 (7.4)	1 (3.7)
**Above the elbow (Trans humeral)**	0	0	0
**At the shoulder (Shoulder disarticulation)**	0	0	0
**Above the shoulder (Forequarter)**	0	0	0
**Total**	14 (51.8)	10 (37.0)	3 (11.1)
Lower (n=15)			
**Foot (including toes or partial foot)**	1 (6.7)	1 (6.7)	0
**At the ankle (Ankle disarticulation) **	0	0	0
**Below the knee (Trans tibial) **	3 (20.0)	7 (46.6)	0
**At the knee (Knee disarticulation) **	0	0	0
**Above the knee (Tran femoral)**	0	1 (6.7)	1 (6.7)
**At the hip (Hip disarticulation)**	1 (6.7)	0	0
**Total **	5 (33.3)	9 (60.0)	1 (6.7)

**Table 2:** The intensity of pain in their residual limbs in landmine explosion survivors under 18 years old

Intensity** (%)**	Distressing n(%)	Discomforting n(%)	Slight Pain n(%)	Total n(%)
**PLPin upper limb amputation**	6 (66.7)	2 (22.2)	1 (11.1)	9 (100)
**PLPin lower limb amputation**	0	6 (85.7)	1 (14.3)	7 (100)

Of the half of subjects who complained of discomforting PLP, only a quarter was incurred upper limb amputation and rest of them had lower limb amputation. Despite current discussion, there have been limited research focused on the subject of PLP and PLS inchildren and adolescents (1, 12-14, 20, 22, 25-27). A review study of children and adolescents with congenital or acquired limb loss emphasized better understanding of the adaptive process in young amputees will be obtained using case-control studies (27). Currently, lack ofsufficient data on subject of phantom pain and sensation in youth amputees have still remained significant. The strength of this study was due to covering all landmine explosion victims less than eighteen years who suffered amputation.

**In conclusion,** phantom phenomena can be experienced by more than one-third of landmine young survivors. We make suggestions for future studies concerning the control and treat phantom pain in children and adolescents amputees. 
